# Novel Pathway for the Combustion Synthesis and Consolidation of Boron Carbide

**DOI:** 10.3390/ma15145042

**Published:** 2022-07-20

**Authors:** Marieta K. Zakaryan, Alina R. Zurnachyan, Narine H. Amirkhanyan, Hasmik V. Kirakosyan, Maksim Antonov, Miguel A. Rodriguez, Sofiya V. Aydinyan

**Affiliations:** 1 Laboratory of Macrokinetics of Solid State Reactions, A.B. Nalbandyan Institute of Chemical Physics NAS RA, P. Sevak 5/2, Yerevan 0014, Armenia; alinaz@ichph.sci.am (A.R.Z.); narineam@ichph.sci.am (N.H.A.); hasmik.kirakosyan@ichph.sci.am (H.V.K.); sofiya.aydinyan@ttu.ee (S.V.A.); 2 Department of Mechanical and Industrial Engineering, Tallinn University of Technology, Ehitajate 5, 19086 Tallinn, Estonia; maksim.antonov@taltech.ee; 3 Instituto de Cerámica y Vidrio, C. Kelsen, 5, 28049 Madrid, Spain; mar@icv.csic.es

**Keywords:** boron carbide, refractory ceramic, self-propagating high-temperature synthesis, combustion synthesis, spark plasma sintering, mechanical properties

## Abstract

A novel pathway for the magnesiothermic reduction of boron oxide and magnesium dodecaboride (MgB_12_) in the presence of carbon by a self-propagating high-temperature synthesis method was proposed that was aimed at the direct preparation of boron carbide nanopowder. The combined utilization of two boron sources, boron oxide and MgB_12_, allowed tailoring the overall caloric effect of the process, increasing the yield of the target product and lessening the laborious leaching process. In addition, it is an alternative way to utilize magnesium borides, which are inevitable side products at boron production. Multivariate thermodynamic calculations performed in the B_2_O_3_-MgB_12_-Mg-C system allowed estimating equilibrium compositions of the products and deducing the optimum composition of the initial mixture for obtaining B_4_C. For the latter, the adiabatic temperature (T_ad_) is 2100 °C, which is theoretically enough for the implementation of the self-propagating reaction. The combustion reaction was shown to be extremely sensitive to the initial mixture composition, external pressure, as well as sample diameter (heat losses). It proceeds in self-oscillatory mode and leads to the product of a layered macrostructure. The combustion product was then consolidated by the spark plasma sintering technique at different conditions. Vickers microhardness was measured, and the wear erosion behavior was examined. The variation in lattice parameters of boron carbide reflected the influence of synthesis, sintering and erosion conditions on the ordering/disordering of the boron carbide structure.

## 1. Introduction

The mechanical properties of B_4_C, in particular fracture toughness, hardness (9.5 on the Mohs hardness scale) and strength combined with low density (2.52 g cm^−3^), are quite exceptional, relying on morphological features and may invariably deviate depending on the preparation pathway [1,2,3,4]. Resistance to fatigue failure is one of the critical design issues for using this material in automotive and aerospace sectors, which will be minimized due to controlling the microstructure of the target material during the synthesis procedure [5,6]. Boron carbide is the third hardest of the technically useful materials after diamond and c-BN. It outperforms other hard materials under high wear and highly abrasive conditions and is considered a promising candidate for wear resistance components, air-jet nozzles, water jet cutting of some refractory ceramics, etc. [7].

Although boron carbide was discovered in the nineteenth century as a by-product of a reaction involving metal borides, methods for its preparation are still being improved. Self-propagating high-temperature synthesis (SHS) [8,9,10] has proven to be a versatile processing route to fabricate refractory ceramics, including but not limited to the B_4_C of diverse morphologies, which exhibit a unique combination of enhanced physicomechanical and tribological properties [11,12,13,14,15]. The short synthesis duration (minutes), the consumption of only the inner heat of reagents, the simplicity of the technological equipment, and the ability to produce high-purity products emphasize the viability of this technology. Wide opportunities for microstructural design and nanomaterials production are at our disposal due to the reasonable selection of the exothermic mixture and macroscopic parameters of the samples as well as control of the macrokinetic aspects of combustion wave propagation [16,17,18,19]. SHS already demonstrated its adaptability not only in the processing of traditional materials but also in the formation of novel structures that are otherwise difficult to produce under conventional conditions [20,21,22,23,24,25,26,27]. The main limitation of SHS processes for the synthesis of advanced materials is the use of only high caloric reactive mixtures enabling to generate, fast enough, a sufficient amount of energy to ensure a self-propagating reaction. For example, the carbothermic reactions of boron oxide or its hydrate are low caloric and have a moderate driving force to propagate in a combustion mode [28,29,30,31]. The direct reaction between boron and carbon is also weak exothermic and has a relatively low enthalpy (ΔH = −71.128 kJ mol^−1^, T_ad_ = 680 °C). In contrary, the magnesiothermic reduction of boron oxide, which is the most common procedure to obtain boron of a purity grade between 90% and 98%, is exothermic (T_ad_ = 2110 °C), but it always leads to a high amount of secondary products, such as MgO, B_2_O_3_ and magnesium borides. The magnesiothermic SHS reduction of boron oxide in the presence of carbon (B_2_O_3_-Mg-C exothermic mixture) was proposed by several authors [32,33,34,35] for the preparation of porous agglomerated particles of B_4_C with a high specific surface area, but the reaction has a low yield (≈19%) and requires long-lasting leaching, the carbide is usually contaminated with magnesium borides formed as stable compounds [36], and the process is very sensitive to carbon impurities [37].

To tackle the challenge, we propose to control the temperature of the B_2_O_3_-Mg-C reaction, increase the yield, lessen the leaching process and create favorable conditions for the decomposition of magnesium borides. Through the utilization of magnesium borides together with boron oxide, more specifically, partial replacement of the raw material, boron oxide, with magnesium dodecaboride (MgB_12_) will allow obtaining the target product. The suggested strategy has also enough perspective to satisfy the global need related to the ethics of reuse of side products of boron and boron carbide on one hand and the quality control thereof on the other, as in the near future, their demand is expected to boost exponentially [38].

Preliminary thermodynamic calculations demonstrated that varying the B_2_O_3_/MgB_12_ ratio in the initial mixture enables covering a wide temperature range of boron carbide preparation; hence, it creates possibilities to contribute to the desired microstructure formation and preservation along with the moderate thermal mode of the combustion wave (controllable temperature, high propagation velocity, high heating and cooling rates). By governing the sizes of ceramic particulates, the improvement in the mechanical performance of the system under study can be achieved.

## 2. Materials and Methods

Boron oxide (ACROS Organics, Thermo Fisher Scientific, Gothenburg, Sweden, particle size µ ≈ 0.1 μm, 98% purity), magnesium dodecaboride (MgB_12_, Pobedit Company, Vladikavkaz, Russia, particle size µ = 0.5–1 μm, 99% purity), magnesium (MPF-3, Ruskhim, Moscow, Russia, particle size 0.15 mm < μ < 0.3 mm, pure grade) and carbon black (P-803, Ivanovo carbon black and rubber JSC, Ivanovo, Russia, particle size µ < 0.1 μm, pure grade) powders were used as raw materials in this research.

The green mixture of the reactants was homogenized in a ceramic mortar for 15 min; cylindrical samples of 50% packing density, height of ≈20 mm, and diameters of 20 and 30 mm were prepared and placed into a stainless-steel Constant Pressure Reactor (CPR-3l). The reactor was sealed followed by evacuation, purged and then filled with argon (purity 99.97%, oxygen content less than 0.02%, Air Products, Moscow, Russia) at the required pressure (0.5−2.5 MPa) to suppress the evaporation of magnesium. The combustion process was initiated with instant heating (18 V, 2 s) of tungsten coil from the upper surface of the sample. Combustion temperature (T_c_, standard error: ±20 °C) and propagation velocity (U_c_, standard error: ±5%) were measured by two C-type tungsten–rhenium (W-5Re/W-20Re) thermocouples 100 μm in diameter. The output signals of thermocouples were transformed by a multichannel acquisition system and recorded by a computer. The maximum combustion temperature for each sample was calculated as an average of the maxima for two temperature profiles. The combustion wave propagation velocity was calculated by dividing the physical distance between the thermocouples by the temporal distance between the thermocouples’ signals (inflection point on each profile). After cooling, the combusted samples were subjected to acid treatment. Hydrochloric acid solution (15 wt %) was used to remove magnesia by-product (MgO) at a bath temperature of 80 °C; then, it was washed with deionized water and dried in a vacuum oven at 100 °C.

To design the composition of the feedstock and optimum conditions for the combustion synthesis of boron carbide, the values of adiabatic combustion temperature (T_ad_) and equilibrium compositions of possible products were calculated by thermodynamic modeling using ISMAN-THERMO software developed for multi-component systems [39].

The characterization of the samples before and after the experiments was accomplished using X-ray diffraction (XRD) analysis. The data on the phase composition were collected by a MiniFlex 600 Rigaku Smart Lab SE diffractometer (Rigaku Corporation, Tokyo, Japan) with a D/teX Ultra 250 1D detector and processed via the JCPDS-ICDD database. The microstructural examinations were performed on the scanning electron microscopes (SEM, FE-SEM, Zeiss Evo MA15, ZEISS, Jena, Germany) equipped with an EDS detector, which measures the energy and intensity distribution of X-ray signals generated by the electron beam interacting with the surface of the specimen. The sintering of the boron carbide powder was carried out by spark plasma sintering (SPS furnace combined with KCE^®^-FCT HP D 10-GB equipment, FCT System GmbH, Rauenstein, Germany) in vacuum, at temperature of 1800–1950 °C with the simultaneous application of 30–50 MPa pressure for a dwell time of 5 and 10 min. The powder was loaded into a 20 mm inner diameter graphite die with a sheet of graphitic paper placed between the punch and the powder, and it was heated up to sintering temperature with a heating rate of 100 °C min^−1^ (cooling rate—200 °C min^−1^). Relative densities of the samples were calculated directly by measuring the density of a sample by Archimedes’ technique (Mettler Toledo ME204, Greifensee, Switzerland) and dividing it by the theoretical density of B_4_C—2.52 g·cm^−3^. The Vickers microhardness (HV5) was measured on the polished surfaces of the samples using a hardness tester 5030 SKV (Indentec, Stourbridge, UK) applying 5 kgf force for 10 s. Values of at least 7 measurements were averaged. Erosive wear testing of materials was conducted with the help of a centrifugal four-channel accelerator detailed elsewhere [40]. The mass of erodent SiO_2_ particles charged into a hopper was 3 kg for running-in period and testing. Silica sand with a particle size of 0.1–0.6 mm was used at the erodent travelling velocity of 50 m s^−1^ under an angle of impingement of 30 °, 45 °, 90 ° at room temperature. Testing of sample 2 was performed only with the angle of 45°, since it demonstrated a high wear rate. The erosion rate was determined as the volume loss of the target sample per mass of erodent particles that attacked it (mm^3^ kg^−1^). The sample was polished by diamond disks down to R_a_ = 0.1 µm before testing. Before the testing, samples were cleaned by acetone, and after testing, they were cleaned by compressed air. The Hardox 400 wear-resistant alloy with a nominal hardness of 400 HBW and yield strength of 1100 MPa (SSAB, Lulea, Sweden) was used as a reference material. Owing to its high toughness, good bendability and weldability, it is used in structures with moderate wear.

## 3. Results and Discussion

### 3.1. Thermodynamic Modeling

The magnesiothermic reduction of boron oxide in the presence of carbon is characterized by high exothermicity (2B_2_O_3_ + 6Mg + C = B_4_C + 6MgO, ΔH = −1124.366 kJ mol^−1^, T_ad_ = 2100 °C) and proceeds in a self-propagating combustion mode.

Several publications have already reported the possibility of synthesis of boron carbide by SHS from boron oxide as a starting material, where the reduction was carried out with magnesium and carbon was added to the mixture as a carburizing agent [32,33,34,35,41,42,43]. To mitigate combustion conditions, namely, to reduce the combustion temperature and to contribute to nanostructure formation, the dilution of the initial mixture by end-products and halides was used [44,45]. The possibility of the preparation of B_4_C from Na_2_B_4_O_7_ + Mg + C by SHS was also reviewed [46]. The borax (Na_2_B_4_O_7_) was chosen as a raw material for its chemical stability. However, it should be noted that in this case, the target material needs to be purified not only by magnesia but also by sodium oxide (Na_2_O). In addition, for the complete interaction, an excess amount of Mg is required to avoid impurities, such as NaBO_2_∙H_2_O, and Mg_3_(BO_3_)_2_. Although the excess of reducer could act as a diluent, nevertheless, here, the excess amount of magnesium increases the exothermicity of the reaction, which, as reported in [47,48], makes it difficult to obtain a fine product with a uniform distribution of particles in the combustion wave.

**Figure 1:**
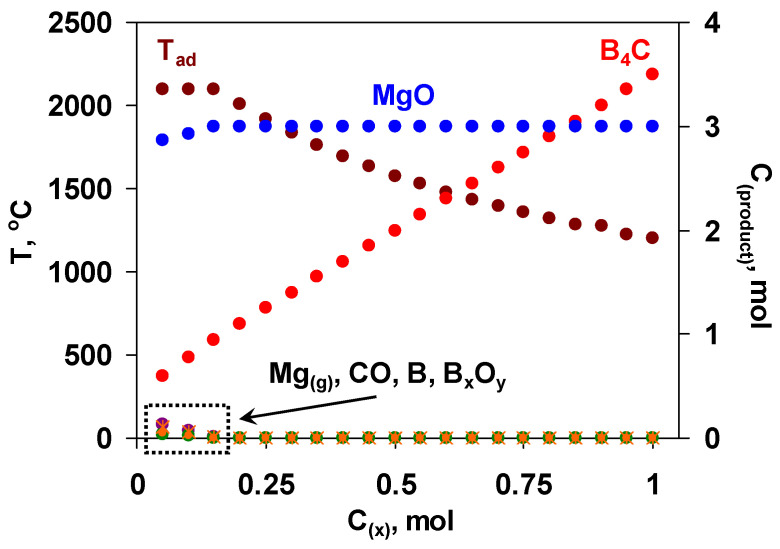
The diagram of the thermodynamic calculations in the B_2_O_3_ + xMgB_12_ + (3x + 0.5)C + (3-x)Mg mixtures; left axis—T_ad_, right axis—MgO, B_4_C, Mg_(g)_, CO, B, B_x_O_y,,_ P = 0.5 MPa.

Hence, the utilization of the mixture of boron oxide and MgB_12_ might serve as an alternative pathway to produce the target boron carbide of reduced grain size, improved purity and increased yield. The use of MgB_12_ has a number of advantages to be detailed below. In particular, when some amount of magnesium and boron oxide is replaced by MgB_12_, the summative reaction is characterized by a comparatively lower exothermicity. As a result, it becomes possible to mitigate and govern interaction conditions as well as control or suppress the evaporation of reactants (especially Mg) that are crucial in terms of both obtaining a fine-grained final product with driven microstructure and more precisely predicting the optimum composition of the green mixture. MgB_12_ serves not only as a source of boron but also as a reducer in active form, which may additionally purify the carbon powder from the adsorbed oxygen [49]. Unlike the furnace methods, where the boron carbide is usually contaminated with magnesium borides, at combustion temperature, magnesium borides decompose and give elemental boron [50]. In addition to all mentioned, the partial substitution of B_2_O_3_ and Mg with MgB_12_ will increase the overall conversion degree and yield of the target product. However, it was not an easy task to approach the thermodynamic optimum of the initial mixture with that complex composition; hence, multivariate calculations were performed with many variables (pressure and composition with four components). The primary calculations were performed at a fixed amount of boron oxide (1 mol) and varying the amount of MgB_12_ (x, mol) based on the following reaction scheme: B_2_O_3_ + xMgB_12_ + (3x + 0.5)C + (3-x)Mg = (3x + 0.5)B_4_C + 3MgO. According to the results obtained (Figure 1), when x < 0.2, some amount of by-products (Mg, B_x_O_y_, B, CO) is present. At that, the adiabatic temperature reaches its maximum value (2100 °C). With increasing the value of x (from 0.2 to 1 mol), the temperature decreases up to 1200 °C, while the yield of boron carbide increases.

Based on the results of thermodynamic analysis, the region of 0.2 ≤ x ≤ 0.3 is favorable for the combustion synthesis of B_4_C in terms of reasonable yield of B_4_C and sufficient exothermicity of the process.

It should be pointed out that for the preliminary prediction, thermodynamic calculations were completed at a pressure of 0.5 MPa. Some calculations were also performed for different pressure values (0.1, 1, 2.5 MPa), demonstrating no significant influence on the phase composition and adiabatic temperature for the mixtures within the thermodynamically optimal range.

### 3.2. Combustion Synthesis of B_4_C

Based on the comprehensive analysis of thermodynamic calculations (Figure 1), combustion experiments were performed with a certain B_2_O_3_ + 0.2MgB_12_ + 2.8Mg + 1.1C mixture. The inert gas pressure and sample diameter were chosen as 2.5 MPa and 30 mm, respectively, as it was not possible to initiate the combustion process of the sample with a smaller diameter (d < 30 mm) at a lower pressure (<2.5 MPa) due to the high volatility of magnesium and heat losses [51].

**Figure 2:**
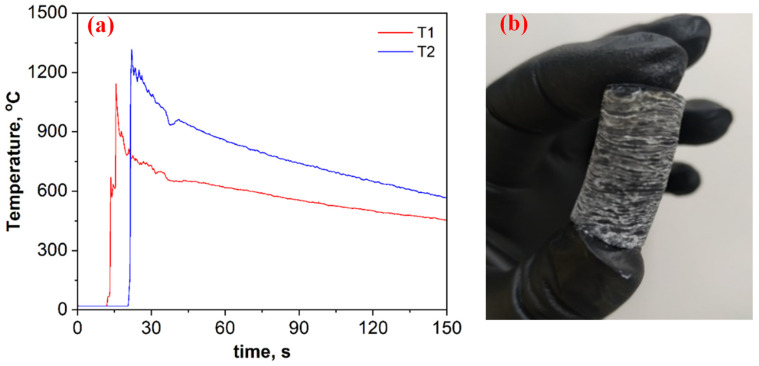
Time–temperature profiles for the B_2_O_3_ + 0.2MgB_12_ + 2.8Mg + 1.1C mixture (**a**) and the overview of the combustion product after SHS (**b**).

Figure 2a depicts time–temperature profiles for the B_2_O_3_ + 0.2MgB_12_ + 2.8Mg + 1.1C mixture. Based on breakthroughs registered on the heating curves (Figure 2a), as well as visual observations of the wave propagation and combustion product (Figure 2b), it is assumed that the self-sustaining reaction proceeds in self-oscillatory mode. After the combustion, the pellet height was almost doubled compared to the initial size, has a layered macrostructure, and was easily divided into thin disks (2−3 mm) (Figure 2b). The heating and cooling rates of the compounds in the combustion wave were calculated from the registered time–temperature profiles (≈1000 °C·s^−1^ and ≈5 °C s^−1^, respectively).

However, the steady-state combustion mode is assumed to be more beneficial from the point of obtaining a uniform product. To convert the self-oscillatory combustion mode of the studied mixture to a steady-state combustion mode, the action of mixture preheating was implemented. Tablets with a diameter of 20 mm were preheated in a constant pressure reactor to a temperature of 450 °C, and a combustion front was initiated (P = 0.5 MPa). Whereas, in the absence of the preheating process, combustion was possible to implement on a sample with a diameter of ≥30 mm at pressure of ≥2.5 MPa. Based on the visual observations, the initial heating in this system led to an increase in the frequency of fluctuations; however, it did not lead to a significant change in the maximum temperature and in the layered structure of the sample.

**Figure 3:**
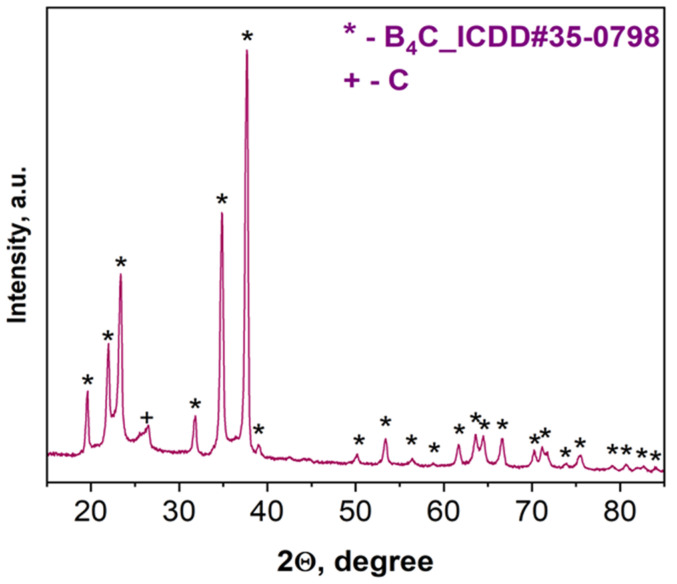
XRD pattern of the combustion product derived from the B_2_O_3_ + 0.2MgB_12_ + 2.8Mg + 1.1C mixture after acid leaching.

According to XRD examinations (Figure 3), the combustion product after acid treatment consists of B_4_C with a trace amount of carbon. Based on the characteristic peaks of the pattern (Figure 3), the average size (20–25 nm) in the vertical direction of crystal for the synthesized boron carbide was calculated (all reflections were considered) according to the Scherrer equation: D = Kλ/βcosθ, where D is the dimension of the crystallite, K is a constant (0.9) and depends on the used peak fitting function, β is the half width at half maximum of the peak, θ is the scattering angle, and λ is the wavelength (0.154 nm) [52].

**Figure 4:**
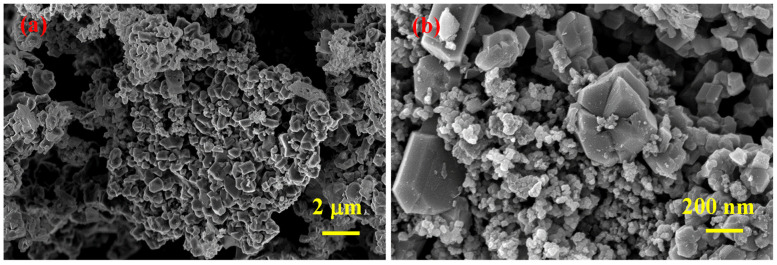
SEM micrographs of SHS-synthesized B_4_C powder after acid leaching at different magnifications (**a**,**b**).

According to SEM analyses results (Figure 4), SHS-synthesized B_4_C (after acid treatment) powder contains agglomerates comprising both small (≈50 nm) and relatively large (<0.5 μm) particles.

SHS-synthesized B_4_C agglomerated powder (after acid leaching) was crushed in a planetary ball mill (at a rotational speed of 500 rpm) for 3 min, using 10 mm in diameter stainless steel balls as crushing–milling media. The obtained powder was sieved and portion, and those with <63 μm particle size were subjected to further examinations.

### 3.3. Spark Plasma Sintering of B_4_C

The consolidation of SHS-derived boron carbide powder was performed at different conditions to determine the influence of consolidation temperature, dwell time, as well as applied pressure on the density, microstructure and properties of the resulting bulk material (Table 1).

According to the results obtained (Table 1), when the consolidation temperature and dwell time are constant and the pressure is increased from 30 to 50 MPa (samples 2 and 4), the relative density of the resulting material increases from 95.6% to 98.8%. When the temperature and pressure are kept constant and the dwelling time is decreased from 10 up to 5 min (samples 3 and 4), the relative density increases from 96․8% to 98․8%. In order to determine the impact of temperature, similar studies were performed by changing the temperature in the range of 1800–1950 °C at a fixed duration and pressure of sintering. The increase in temperature from 1800 to 1950 °C increased the relative density by 16.3% (samples 1, 3 and 5). From all the above-mentioned results, it becomes obvious that relatively high temperatures and pressures with moderate dwell time are favorable for the sintering of SHS synthesized fine B_4_C powder. The dwell time impact can be conditioned by the fact that carbon can be dissolved in B_4_C and form carbon-rich carbides [13,53,54]. Thus, the sample with maximum relative density was obtained (99.2%) at 1950 °C temperature, and the dwell time was 10 min at a pressure of 50 MPa. This value is commensurate or even exceeds a number of relative density values for the SPSed samples reported in the literature, even considering stricter consolidation conditions used in those works [55,56,57].

Table 1 shows the Vickers microhardness values measured for the samples with the relatively high-density values (samples 3, 4 and 5). As expected, the densest specimen is characterized by the highest hardness (HV5 = 2641 ± 39), which is comparable with the data reported in the literature [36,49].

**Figure 5:**
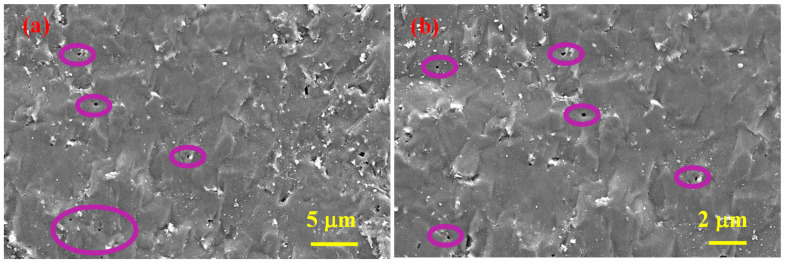
SEM micrographs of the polished surface of SHS-synthesized B_4_C after SPS consolidation at two magnifications (**a**,**b**), sample 5.

**Figure 6:**
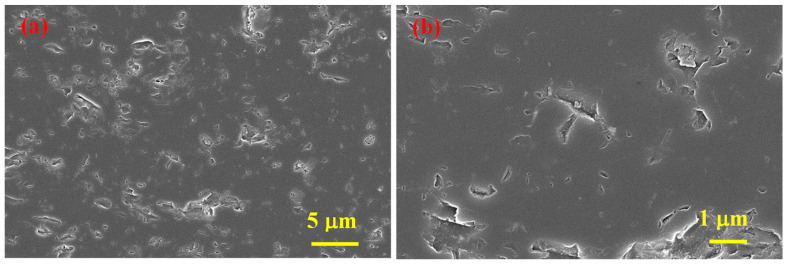
SEM micrographs of the vertical cross-section of SPS consolidated B_4_C at two magnifications (**a**,**b**), sample 5.

Figure 5 depicts SEM micrographs of SHS-synthesized B_4_C after SPS consolidation (sample 5), and Figure 6 depicts SEM micrographs of the cross-section of the same sample, where the observed spherical pores of nanometer to submicron size indicate the presence of a gas (absorbed oxygen considering the nature of precursors) of some type and the subsequent release thereof during the sintering (purple circles).

**Figure 7:**
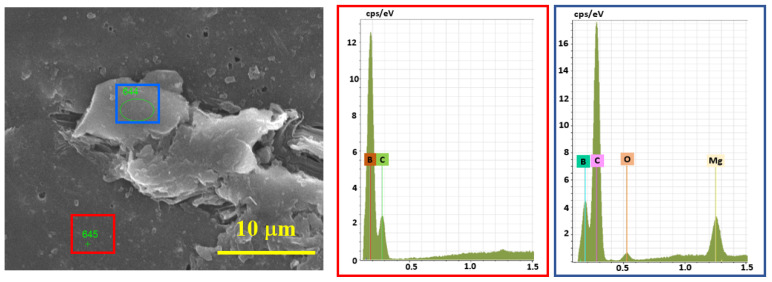
EDS analysis results of the cross-section of SPS-consolidated B_4_C, sample 5.

According to EDS analysis (Figure 7), the presence of Mg with a trace amount of O (from the oxidized surface of Mg) was observed in the under-sintered areas and next to the pores, and in the bulk parts, only the signals of boron and carbon were present. Therefore, the full densification of B_4_C is assumed to be prevented by an insignificant amount of Mg.

Some additional experiments were performed to demonstrate the pre-pressing influence on the sinterability of powders. For that purpose, SHS-produced powders were preliminary cold pressed at 150 MPa pressure. After that, the sintering was carried out at two different temperatures (1800 °C and 1900 °C) at a fixed dwell time and pressure (10 min and 50 MPa, respectively) (Table 2).

According to the results obtained (Table 1 and Table 2), when the consolidation is carried out at 1800 °C (10 min dwell time, 50 MPa pressure), the relative density of pre-pressed samples increases by 15.2% (samples 1 and 6), and at 1900 °C, it increases only by 2.3% (samples 3 and 7). Hence, the cold pre-pressing of samples had a significant influence on the sintering behavior and allowed achieving high-density samples at relatively lower temperatures.

A comparative analysis demonstrated the privilege of SPS densification of SHS-derived B_4_C nanopowders in comparison to B_4_C of wide particle size distribution (from 100 nm to several micrometers) produced from elemental powders [58]. On another hand, the densification behavior and mechanical properties of sub-micrometer sized commercial boron carbide (with 2−3 μm average grain size) were comparable to SHS-produced B_4_C regardless of the difference in sintering conditions [59,60]. B_4_C obtained by a carbothermic reduction of boron oxide/boric acid allowed achieving a similar densification value when sintered at higher temperature (2100 °C), as it comprised particles of up to 5 μm [61]. Commercially available B_4_C nanopowder (591 ± 26 nm) was possible to sinter at a comparatively lower temperature but at higher pressure [62]; however, it was not superior to the SHS-produced B_4_C in terms of mechanical performance. Microwave-synthesized B_4_C comprising 50−300 nm sized particles after the SPS densification at similar conditions demonstrated a similar relative density value but higher microhardness [49]. Usually, higher hardness values are ascribed to higher sintering temperatures due to the dependence of diffusion to sintering temperature when a denser structure is achieved. However, at the same relative density values, higher hardness is attributed to the finer particles or so-called preserved nanostructure at the fast SPS processing, which is apparently influenced by the synthesis method of nanopowder and holds the key of the origin of the structure–property characteristics of the compacted samples.

**Figure 8:**
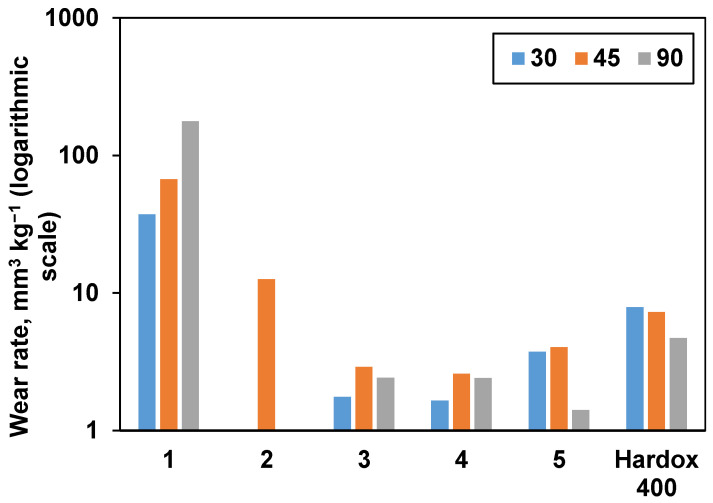
The influence of impact angle on the wear rate of materials. Hardox 400 wear-resistant alloy was used as a reference.

**Figure 9:**
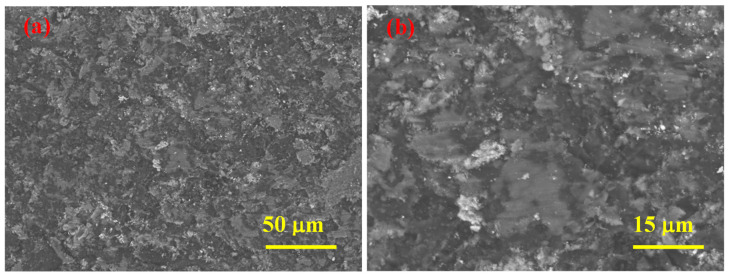
SEM micrographs at two magnifications of sample 5 (SPS-consolidated B_4_C) taken after the erosion test at impact angle of 30 ° (**a**, **b**).

Consolidated samples were further subjected to erosive wear testing. Hardox 400 reference wear-resistant alloy demonstrated behavior typical for ductile materials with the highest wear rate under a low angle of impact (30 °). The results are presented in Figure 8, and SEM images taken after wear erosion testing are illustrated in Figure 9. From Figure 8, it is obvious that samples 1 and 2 had the highest wear rate under a normal impact angle (90 °), as it was expected from their densification behavior (they exhibit the lowest relative density and Vickers hardness values). Samples 3 and 4 demonstrated mixed behavior with maximum wear at 45 °. Unexpectedly, sample 5 had a very low wear under normal impact conditions, which is more typical for ductile materials. When comparing the tests performed at the highest studied angle (90 °), sample 5 showed the lowest volumetric wear rate (1.41 mm^3^ kg^−^^1^) among the all studied samples, which is commensurate with the wear rate values of similar materials reported in the literature [7,49].

Regardless of the sintering temperature, the main erosive wear mechanisms operating at given conditions are similar, i.e., fracturing and/or fragmentation of carbide and removal of the whole ceramic grains due to intergranular cracking combined with some plastic deformation. From the micrographs (Figure 9), it can be seen that the appearance of the eroded worn surface of sample 5 shows obviously smaller pits and scratches on the sample sintered at higher temperature, while additionally, the plastic deformation (smearing of material) is present.

**Figure 10:**
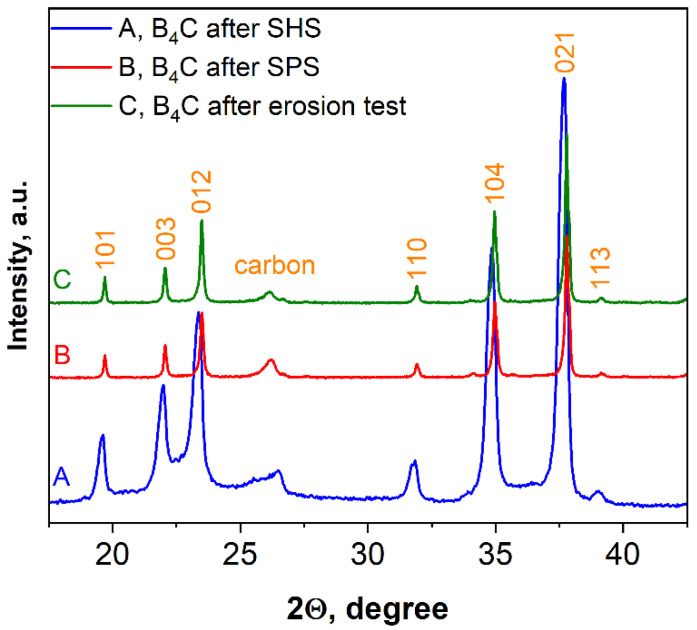
XRD patterns of boron carbide, sample 5.

Figure 10 shows the XRD pattern of SHS-synthesized B_4_C (pattern A) as well as the patterns of the bulk sample obtained after SPS consolidation (pattern B) and the same sample after the erosion test (pattern C). According to the calculations by using the Scherrer equation, an increase in average size in the vertical direction of crystal after SPS was observed (50–75 nm or two to three times higher compared to the SHS produced powder).

As can be seen from Figure 10, there is some displacement of the boron carbide peaks, which, naturally, may lead to changes in the lattice parameters of the material.

Table 3 shows the parameters of the crystal lattices calculated on the basis of these three XRD patterns (B_4_C-ICDD#35-0798 is presented as a reference).

Comparing the lattice parameters of boron carbide, one can observe a variation in the values of a and c, thus reflecting the influence of synthesis, sintering and erosion conditions on the boron carbide structure. The increased a and c values of SHS powder are a result of limits for the replacement of boron atoms by carbon and relative disorder due to the high cooling rate of the combustion process, but they are notably lower compared to the literature data, which can be associated with the better crystallization conditions provided by the SHS process at a moderate temperature [44,54]. During SPS, the process of crystallization and lattice ordering continues, which is accompanied by a decrease in the lattice parameters.

## 4. Conclusions

- B_4_C powder was produced by the novel pathway of magnesiothermic reduction of (B_2_O_3_ + MgB_12_) mixture in the presence of carbon.
- The utilization of magnesium dodecaboride as an alternative source of B and Mg demonstrated several advantages in terms of controlled self-sustained combustion reaction, desired microstructure formation, less laborious acid-leaching process and absence of impurities, such as other borides.
- Multivariate thermodynamic calculations allowed revealing the optimum composition of the initial mixture (B_2_O_3_ + 0.2MgB_12_ + 2.8Mg + 1.1C) beneficial for the self-propagating combustion reaction and target material preparation.
- The critical influence of sample diameter, inert gas pressure and preheating temperature was demonstrated.
- Self-oscillatory mode of the combustion was registered, leading to the formation of a product with a layered structure comprising agglomerates of particles of different origin (small (≈50 nm) and relatively large (<0.5 μm) particles).
- The obtained powder was successfully consolidated by the SPS method. A compact sample with a density of 99.2% was obtained at 1950 °C, for which the measured Vickers microhardness value (2641 ± 39 HV5) and wear erosion behavior are commensurate to the data reported in the literature.

## Figures and Tables

**Table 1:** Consolidation conditions and properties of bulk boron carbide.

N°	T, °C	t, min	P, MPa	Relative Density, %	HV5
1	1800	10	50	82.9	-
2	1900	5	30	95.6	-
3	1900	10	50	96.8	2416 ± 36
4	1900	5	50	98.8	2152 ± 32
5	1950	10	50	99.2	2641 ± 39

**Table 2:** Consolidation conditions and properties of preliminary cold-pressed bulk boron carbide.

N°	T, °C	t, min	P, MPa	Relative density, %
6	1800	10	50	98.1
7	1900	10	50	99.1

**Table 3:** Lattice parameters for boron carbide (calculated for hkl = 021 peak).

Sample	a	b	c
B_4_C-ICDD#35-0798 standard	5.60	5.60	12.08
B_4_C powder after SHS	5.62	5.62	12.14
B_4_C after SPS consolidation	5.61	5.61	12.11
B_4_C consolidated sample after erosion test	5.56	5.56	12.11

## Data Availability

Not applicable.
